# Acetylsalicylic acid enhance tolerance of *Phaseolus vulgaris* L. to chilling stress, improving photosynthesis, antioxidants and expression of cold stress responsive genes

**DOI:** 10.1186/s40529-018-0222-1

**Published:** 2018-02-15

**Authors:** Mona H. Soliman, Aisha A. M. Alayafi, Amr A. El Kelish, Abdelghafar M. Abu-Elsaoud

**Affiliations:** 1 Biology Department, Faculty of Science, Taibah University, Al-Sharm, Yanbu El-Bahr, 46429 Kingdom of Saudi Arabia; 2 Botany and Microbiology Department, Faculty of Science, Cairo University, 12613 Giza, Egypt; 3 Biological Sciences Department, Faculty of Science, University of Jeddah, Jeddah, Kingdom of Saudi Arabia; 4 Botany Department, Faculty of Science, Suez Canal University, Ismailia, Egypt

**Keywords:** Chilling stress, Acetylsalicylic acid, ASA, Antioxidants enzymes, Superoxide dismutase, Catalase, Peroxidase

## Abstract

**Background:**

High and low temperatures constitute the most damaging type of abiotic stress and limit the survival, and productivity of plants. The present study aimed to evaluate the role of exogenous applications of acetylsalicylic acid (ASA) in reducing the deleterious effects of cold stress. *Phaseolus vulgaris* L. seedlings were treated with foliar-sprayed ASA at concentrations of 0–3 mM and then subjected to chilling stress at 4 °C for 2 or 4 days.

**Results:**

Growth, photosynthesis, biochemical alterations, oxidative damage and antioxidant enzyme activities as well as the expression of cold-responsive genes (CBF3–COR47), were monitored during the experiment. ASA applications substantially improved several growth and photosynthetic parameters, including shoot biomass, dry weight, and photosynthetic pigments, of *P. vulgaris* seedlings exposed to different durations of chilling stresses. The ASA foliar spray treatments significantly (*p* < 0.05) rescued the growth and photosynthetic pigments of *P. vulgaris* seedlings under different chilling stresses. The total soluble sugars markedly increased during 0–4 days of chilling stress following ASA foliar spraying. The exogenous application of ASA significantly (*p* < 0.05) increased the accumulation of proline in *P. vulgaris* seedlings under chilling stress. At the gene expression level, ASA significantly (*p* < 0.05) upregulated the cold-responsive genes CBF3 and COR47.

**Conclusions:**

As a result, we speculate that, the application of exogenous ASA alleviated the adverse effects of chilling stress on all measured parameters, and 1 and 2 mM ASA exhibited the greatest effects.

## Background

Climate change and global warming generate different kinds of biotic and abiotic stresses that in turn alter plant responses at the transcriptomic, proteomic and metabolomic levels (Chartzoulakis and Psarras 2005; Khan and Khan 2013; El Kelish et al. 2014). Moreover, climate change severely disturbs the biochemistry, quantity, and quality of crop yields (Jaleel et al. 2009; Miller et al. 2010; Cramer et al. 2011; Pereira 2016). Environmental challenges lead to a wide range of responses in plants, such as morphological, physiological, and molecular changes (Zandalinas et al. 2017). According to the Food and Agriculture Organization (FAO), the population is increasing rapidly and will reach approximately 10 billion in 2050. Moreover, food production is decreasing because of various abiotic stresses. As such, the earth will require 70% more food by 2050 (Mahajan and Tuteja 2005; Gill and Tuteja 2010). Therefore, it is necessary to obtain crops that display both enhanced vigor and tolerance to various environmental factors to cope with the future problem of food security (Bita and Gerats 2013).

Chilling injury can occur at temperatures between 0 and 15 °C (Kratsch and Wise 2000; Allen and Ort 2001). Various phenotypic symptoms of plants in response to chilling stress are clear, including reductions in leaf chlorophyll content, wilting, chlorosis, and necrosis. Moreover, different metabolic modifications are induced, such as reductions in unsaturated fatty acid contents and increases in the permeability of cell membranes, which collectively reduce plant performance (Bracale and Coraggio 2003; Tong et al. 2005; Miura and Furumoto 2013). One of the essential cellular response throughout a freeze–thaw cycle is the maintenance of plasma membrane integrity. This maintenance reduces the efficiency of photosystem II (PSII); damages photosystem I (PSI); alters the carbon reduction cycle, CO_2_ assimilation, and photosynthetic pigment complex systems; and accelerates reactive oxygen species (ROS) formation (Guy 1990; Thomashow 2001; Allen and Ort 2001; Saibo et al. 2009).

Beans (*Phaseolus lunatus*), corn (*Zea mays*) and tomato (*Solanum lycopersicum*) are considered relatively sensitive to environmental stress. Many studies have reported the impact of chilling injury and how short exposure to temperatures below freezing can destroy whole crops (Kratsch and Wise 2000; Chinnusamy et al. 2007). Most plants have developed numerous systems to manage performance during environmental stress. For instance, to minimize chilling-induced injury, plants can upregulate different scavenging systems, such as enzymatic antioxidants and non-enzymatic metabolites (Gill and Tuteja 2010). Moreover, plants can synthesize plant growth regulators [salicylic acid (SA)] and osmoprotectants (proline) (Gautam and Singh 2009; AbdElgawad et al. 2016; Tabassum et al. 2017). Most of these compounds shield membranes and the photosynthetic apparatus from the harmful effects of environmental stress (Foyer and Noctor 2003).

Among the best-recognized gene family is the CBF/DREB1 family, whose members play a major role in chilling tolerance in Arabidopsis (Thomashow 1998, 2001). CBF transcription factors act as a regulatory hub for low-temperature acclimation, controlling the expression of cold-regulated (COR) target genes that include CRT/DRE *cis*-elements in their promoters (Thomashow 2010). The overexpression of CBF leads to an intense activation of COR genes and successively enhances chilling and dehydration tolerance in sensitive plants (Kasuga et al. 1999; Jaglo et al. 2001). Furthermore, the constitutive overexpression of Arabidopsis CBF1/DREB1b confers chilling stress tolerance to cucumber and potato (Gupta et al. 2012; Movahedi et al. 2012; Caffagni et al. 2014). Thus, CBF/DREB1 is the primary and most critical regulatory gene associated with chilling stress in plants.

SA and ortho-hydroxybenzoic acid are essential signaling phenolic compounds associated with plant development and tolerance to abiotic and biotic stresses (Raskin 1992; Khan et al. 2003, 2012). SA is involved in the adjustment of vital plant physiochemical activities, such as the light and dark reactions, carbon–nitrogen metabolism, proline metabolism, and ROS scavenger systems, and therefore offers protection against abiotic stresses in plants (Khan et al. 2003; Saleh et al. 2007; Simaei et al. 2012; Miura and Tada 2014; Ruelland 2017). Aspirin, a trade name for acetylsalicylic acid (ASA), can be obtained by the spontaneous hydrolysis of SA (Senaratna et al. 2000). Similarities between the chemical, physical, and physiological characteristics of ASA and SA have encouraged plant scientists to use the former in biological experiments (Kupferwasser et al. 1999; Canakci and Munzuroğlu 2007).

The objective of this study was to evaluate the role of exogenous ASA in rescuing *Phaseolus vulgaris* from the deleterious effects of chilling stress. The exogenous application of ASA may represent a strategy to increase plant tolerance to chilling stress by regulating antioxidant defense systems and increasing the levels of key metabolites and genes involved in chilling stress tolerance.

## Methods

### Plant materials and ASA treatment

A greenhouse experiment was conducted at the Department of Botany of the Faculty of Science, Suez Canal University, Ismailia, Egypt, during November and December 2016. Seeds of common white bean (*P. vulgaris* L.) were purchased from the Department of Vegetable Crop Research of the Agricultural Research Center, Giza, Egypt. Before they were sown, *P. vulgaris* seeds were superficially sterilized with 5% sodium hypochlorite, rinsed thoroughly with distilled water and then dried on filter paper for 30 min. Pure crystals of ASA (99.5%) (Sigma Chemical Company, USA) was prepared at five concentrations (0.1, 0.5, 1, 2 and 3 mM) in addition to a non-ASA control. The ASA crystals were dissolved in 0.1 mL of ethanol (95%). NaOH and HCl were used for neutral pH adjustments. Five sterilized *P. vulgaris* seeds were planted in plastic pots (20 cm in length × 15 cm in diameter) filled with a clay: sand mixture (1:1, w/w). Germination was carried out for 30 days under normal greenhouse conditions of 25 ± 4.0 °C and a 16-h photoperiod. Once they had emerged, the seedlings were irrigated weekly with tap water to field capacity. Thirty-day-old seedlings were subjected to foliar sprays of ASA at concentrations of 0.1, 0.5, 1, 2 or 3 mM concentrations. The foliar sprays were applied using a hand sprayer until the droplets began to run off, after which the seedlings were allowed to grow for 24 h. The non-ASA control treatment was sprayed with distilled water only, and there were three replicates of each treatment as well as the control.

### Chilling stress experiments

For chilling stress treatments, 24 h after ASA spraying, the seedlings were thinned to three seedlings pot^−1^, after which the pots were transferred to a 2 °C cold room that contained metal halide lamps that generated 250 μmol m^−2^ s^−1^ of photosynthetic photon flux density (PPFD) under a 12-h photoperiod. The seedlings remained under cold-shock conditions at 4 °C for 2 or 4 days under 16-h photoperiod and a PPFD of 300 μmol m^−2^ s^−1^. Each treatment consisted of three replicates in a randomized complete block design. Fresh samples were taken after 0, 2 and 4 days of chilling for morphological and biochemical analyses. The remaining samples were collected and stored at − 80 °C until use.

### Determination of photosynthetic pigments and soluble sugars

The photosynthetic pigments [chlorophyll a (chl. a) and chlorophyll b (chl. b)] were determined in accordance with the spectrophotometric method (Jenway UV/Vis spectrophotometer, UK) recommended by Holder (1965). The absorbance was measured against a blank sample of pure acetone (85%) at 2 wavelengths: A645 and A663 (nm). The pigment fractions (chl. a and chl. b) were expressed as milligrams per gram of dry weight (DW) (Strain and Svec 1966).

The procedures for the extraction of the total soluble sugars from *P. vulgaris* leaves were described by Ciha and Brun (1978). The total soluble sugars were quantified using a modified anthrone acid assay (Irigoyen et al. 1992).

### Determination of protein, amino acid, and proline contents

The total soluble protein content in the fresh leaves of *P. vulgaris* was determined as described by Bradford (1976) using bovine serum albumin (BSA) as a standard. The total free amino acid content was determined as described by Dubey and Rani (1989), with modifications. A 0.2-g sample of dried leaves was grinded in 10 mL ethanol (80%). After filtration, one hundred microliters of the extract and 5 mL of ninhydrin reagent were mixed, after which the mixture was shaken gently and then heated for 10 min in a boiling water bath. Subsequently, the mixture cooled, its absorbance was measured spectrophotometrically at 570 nm. The extraction of proline was carried out with aqueous sulfosalicylic acid, and the proline contents were determined spectrophotometrically in accordance with the methods of Sadasivam (1992). The proline content in each sample was measured in milligrams per gram of fresh weight (FW) and determined using a standard curve of analytical-grade proline.

### Cellular lipid peroxidation

Lipid peroxidation was investigated via the malondialdehyde (MDA) content, which was estimated spectrophotometrically using thiobarbituric acid (TBA)-MDA (TBA-MDA) assays (De Vos et al. 1991). The extraction of lipid peroxides was carried out using 500 mg of fresh shoot tissue, 0.3 mL of 10% trichloroacetic acid, and 1 mL of 0.5% TBA. The TBA-chromogen color was measured spectrophotometrically at 532 nm.

### Determination of ascorbic acid (AA) content

The AA content was measured by redox titration using iodine solution in accordance with the methods of McHenry and Graham (1935).

### Determination of antioxidant enzymes

Enzyme extracts were prepared by homogenizing 1 g of fresh leaves of *P. vulgaris* with 5 mL of cold phosphate buffer (pH = 7.0). The extracts were then centrifuged at 18,000*g* for 30 min at 4 °C, after which the supernatant was filtered and stored at − 20 °C for further enzyme assays.

The ascorbate peroxidase (APX) activity was determined as described by Rao et al. (2007). The APX activity was recorded by following the decrease in absorbance at 290 nm for 3 min in 1 mL of a reaction mixture that contained 100 mM phosphate buffer (pH 7.5), 0.5 mM ascorbate, 0.2 mM H_2_O_2_ and 30 μL of enzyme extract. The enzyme activity was expressed as moles of oxidized ascorbate per minute per mg of protein.

The catalase (CAT) activity was determined by recording at 240 nm the consumption of H_2_O_2_ for 30 s in 3 mL of a reaction mixture that consisted of 100 mM phosphate buffer (pH 7.0), 20 μL of 30% H_2_O_2_ and 30 μL of enzyme extract (Aebi 1984).

The activity of peroxidase (POD) was determined as described by Jiang et al. (2002). The reaction mixture consisted of 1 mL of enzyme extract and guaiacol as a substrate. A 3-mL reaction mixture consisted of 100 mM sodium phosphate buffer (pH 7.0) and 20 mM guaiacol. The increase in absorbance at 470 nm during a 3-min period was measured spectrophotometrically after 20 μL of H_2_O_2_ was added to the mixture. The enzyme activity was defined as the change in the optical density per mg of proteins per minute.

The activity of superoxide dismutase (SOD) was determined photochemically in accordance with the methods of Giannopolitis and Ries (1977). The assays were carried out under illumination. One unit of SOD activity was defined as the amount of enzyme required to inhibit 50% of a nitro blue tetrazolium (NBT) chloride reaction, which was recorded at 560 nm.

### Gene expression of cold-responsive genes (CBF3 and COR47)

#### RNA extraction and first-strand cDNA synthesis

RNA extraction was carried out by a QIAGEN RNA extraction kit (QIAGEN, Germany). DNA digestion was performed with DNaseI (QIAGEN, Germany). The RNA yield and quality were determined using a NanoDrop system (ND-1000; Thermo Fisher Scientific). First-strand cDNA was synthesized from 1 μg of high-quality total RNA using SuperScript II Reverse Transcriptase in accordance with the manufacturer’s instructions (QIAGEN, Omniscript RT).

#### Real-time PCR analysis

The specific primer sequences of two cold-responsive genes, CBF3 and COR47, were used (Table 1), and the β-actin gene was used as an internal standard. The obtained cDNA was used in qRT-PCR, which was performed in a 20-μL reaction mixture that contained one unit of Taq polymerase (Fermentas, Canada), 3.5 mM of MgCl_2_, 1 unit of PCR buffer, 0.5 mM of each primer, 10 mM dNTPs, 0.5 units of SYBR Green I, 0.6 mL of DMSO (Sigma-Aldrich), and 2 μL of template cDNA, using a Roter-Gene Real-Time thermocycler (QIAGEN, USA). The PCR conditions were as follows: 95 °C for 1 min, followed by 40 cycles of 95 °C for 15 s, 58–60 °C for 30 s and 72 °C for 30 s. The relative expression of the cold-responsive genes (CBF3 and COR47) was calculated using the 2^−ΔΔCt^ comparative CT method (Livak and Schmittgen 2001). Differences in the CBF3 and COR47 transcriptional patterns in response to different ASA concentrations were analyzed using SPSS 16.0 software. Statistical significances were determined using one-way analysis of variance (ANOVA) and post hoc Duncan multiple range tests (DMRTs). Significance was established at *p* < 0.05. The means and standard deviations were calculated from experiments performed in triplicate and are presented as n-fold differences in expression.

**Table 1:** The sequences of the primers used in qRT-PCR

Primer name		Primer sequence (5_–3_)	Annealing temperature (°C)
CBF3	F R	5′-TTCCGTCCGTACAGTGGAAT-3′ 5′-AACTCCATAACGATACGTCGTC-3′	58
COR47	F R	5′-CGGTACCAGTGTCGGAGAGT-3′ 5′-ACAGCTGGTGAATCCTCTGC-3′	58
β-Actin	F R	5′-CGCGACCTCACAGACTACCTG-3′ 5′-CGTAGGACTTCTCCAGGGAGC-3′	58

### Statistical analysis

The data obtained from various growth, photosynthetic, and biochemical parameters were analyzed statistically Using IBM-SPSS 23.0 statistical software for Mac OS. Two-way ANOVA was performed to assess differences between various chilling stress levels and ASA concentrations. All comparisons were made at a probability level of 95% (*p* ≤ 0.05). The means are the average of three replicates, and DMRTs were used for comparing means following the 2-way ANOVA.

## Results

The seedlings of *P. vulgaris* grown under normal conditions (non-stressed) and treated with foliar sprays of different concentrations of ASA (0 to 3 mM ASA) exhibited no significant differences in various growth parameters (*p* > 0.05), especially shoot biomass (g plant^−1^), root biomass (g plant^−1^), the shoot:root ratio (g g^−1^), chl. a (mg g^−1^ FW), chl. b (mg g^−1^ FW), and total chlorophyll (mg g^−1^ FW) (Table 2). However, *P. vulgaris* seedlings exposed to chilling stress at two levels (2 or 4 days) and seedlings not treated with ASA foliar sprays showed general deleterious effects on growth parameters (Table 2). The shoot biomass decreased significantly (*p* < 0.05) from 2.85 ± 0.002 g before chilling stress to 1.67 ± 0.03 and 0.094 g plant^−1^ after 2 and 4 days of chilling stress, respectively. Likewise, the shoot DW significantly decreased from 1.65 ± 0.001 g plant^−1^ in the non-stressed control plants to levels of 0.064 ± 0.01 and 0.55 ± 0.00 g plant^−1^ in *P. vulgaris* seedlings exposed to chilling stress for 2 and 4 days, respectively. The photosynthetic pigments showed a similar damaging pattern under chilling stress, e.g., the chl. a content significantly decreased from 6.95 ± 0.01 mg g^−1^ FW in the non-stressed control seedlings to 4.86 ± 0.00 and 4.75 ± 0.02 mg g^−1^ FW in the seedlings exposed to 2 and 4 days of chilling stress (Table 2).

**Table 2:** Shoot fresh weight (g plant^−1^), dry weight (g plant^−1^), shoot: root ratio (g g^−1^), chlorophyll-a (mg g^−1^ FW), chlorophyll-b (mg g^−1^ FW), chlorophyll a/b, and total chlorophyll contents (mg g^−1^ FW) of *P. vulgaris* L. after foliar application of different concentration of acetylsalicylic acid (ASA; 0, 0.1, 0.5, 1, 2, 3 mM) and exposed to different level of chilling stress (0, 2, 4 days)

Chilling (d)	ASA foliar spray (mM)	Shoot FW (g plant^−1^)	Shoot DW (g plant^−1^)	Shoot: root ratio (g g^−1^)	Chl-a (mg g^−1^ FW)	Chl-b (mg g^−1^ FW)	Chl a/b	Total chlorophyll (mg g^−1^ FW)
0	0	2.85 ± 0.002 g	1.65 ± 0.00ij	0.78 ± 0.09i	6.95 ± 0.01 h	3.75 ± 0.03de	1.85 ± 0.01i	10.70 ± 0.04a
	0.1	2.87 ± 0.02 g	1.64 ± 0.00i	0.69 ± 0.01i	6.92 ± 0.00 h	3.74 ± 0.02de	1.85 ± 0.01i	10.67 ± 0.02a
	0.5	2.85 ± 0.01 g	1.66 ± 0.02ij	0.68 ± 0.00i	6.95 ± 0.01 h	3.76 ± 0.02de	1.85 ± 0.00i	10.71 ± 0.03a
	1	2.85 ± 0.01 g	1.66 ± 0.00ij	0.70 ± 0.01i	6.93 ± 0.00 h	3.71 ± 0.00def	1.87 ± 0.00i	10.64 ± 0.00a
	2	2.85 ± 0.00 g	1.64 ± 0.02ij	0.68 ± 0.01i	6.96 ± 0.03 h	3.72 ± 0.00ef	1.87 ± 0.01i	10.68 ± 0.03a
	3	2.85 ± 0.00 g	1.72 ± 0.05j	0.70 ± 0.01i	6.96 ± 0.02 h	3.72 ± 0.00ef	1.87 ± 0.01i	10.68 ± 0.02ab
2	0	1.67 ± 0.03bc	0.64 ± 0.01b	2.25 ± 0.05b	4.86 ± 0.00b	2.68 ± 0.00d	1.82 ± 0.00b	7.54 ± 0.00bc
	0.1	1.81 ± 0.04bcd	0.68 ± 0.00bc	1.75 ± 0.35bc	4.92 ± 0.00c	2.97 ± 0.00b	1.66 ± 0.00c	7.88 ± 0.00c
	0.5	1.92 ± 0.01d	0.86 ± 0.00d	1.85 ± 0.28ef	5.13 ± 0.00 fg	3.34 ± 0.00a	1.54 ± 0.00 fg	8.48 ± 0.00c
	1	2.11 ± 0.08e	1.25 ± 0.01 g	1.39 ± 0.01f	5.22 ± 0.01de	3.18 ± 0.00b	1.64 ± 0.00ef	8.40 ± 0.01a
	2	1.82 ± 0.03 cd	0.73 ± 0.01c	1.48 ± 0.00f	5.18 ± 0.00d	3.12 ± 0.10b	1.66 ± 0.05de	8.29 ± 0.10a
	3	1.73 ± 0.05bc	0.83 ± 0.01d	1.86 ± 0.02de	5.07 ± 0.05c	2.95 ± 0.02c	1.72 ± 0.03c	8.02 ± 0.03a
4	0	0.94 ± 0.03a	0.55 ± 0.00a	2.56 ± 0.01a	4.75 ± 0.02a	2.37 ± 0.00 g	2.01 ± 0.01a	7.12 ± 0.01a
	0.1	1.79 ± 0.01bcd	0.71 ± 0.00bc	1.69 ± 0.01 cd	4.98 ± 0.01def	3.22 ± 0.01a	1.55 ± 0.00d	8.21 ± 0.02a
	0.5	2.17 ± 0.01e	1.16 ± 0.01f	1.59 ± 0.04 h	5.53 ± 0.01 g	3.41 ± 0.01b	1.62 ± 0.01 h	8.94 ± 0.00ab
	1	2.48 ± 0.02f	1.72 ± 0.01j	0.88 ± 0.01 h	5.74 ± 0.02efg	3.28 ± 0.00c	1.75 ± 0.01 h	9.02 ± 0.01bc
	2	2.25 ± 0.00e	1.44 ± 0.01 h	1.48 ± 0.01 h	5.70 ± 0.01c	2.86 ± 0.00 g	2.00 ± 0.00 fg	8.56 ± 0.01c
	3	1.66 ± 0.01b	0.97 ± 0.02e	1.77 ± 0.06 h	5.64 ± 0.00c	2.95 ± 0.01f	1.91 ± 0.00 g	8.59 ± 0.00c
Univariate; two-way ANOVA								
F_(corrected)_		423.8***	1013.3***	2559.6***	263.5***	85.6***	1768.3***	34.23***
F_(intercept)_		111,905***	131,774***	2,011,183***	286,459***	239,993***	1,652,469***	622.28***
F_(ASA)_		2540.9***	5482.6***	20,123***	1506.7***	232.4***	13,321.5***	68.25***
F_(chilling)_		203.1***	686.9***	353.3***	169.7***	104.2***	395.9***	4.87**
F_(ASA*chilling)_		110.8***	282.7***	149.9***	61.9***	46.95***	143.9***	42.11***

Data represented are mean of three points ± standard deviation

* Significant at p < 0.05; ** *p* < 0.01; *** *p* value < 0.001, Variations between different chilling stresses and foliar ASA concentration were assessed by univariate analyses followed by Duncan’s test statistic. Means with the same letters are not significantly different according to Duncan’s multiple comparisons

The application of ASA foliar sprays significantly improved various growth parameters of *P. vulgaris* seedlings exposed to different durations of chilling stress (Table 2). The shoot biomass of *P. vulgaris* seedlings subjected to 2 days of chilling stress significantly (*p* < 0.05) increased from 1.67 ± 0.03 g in the non-ASA-treated seedlings to 2.11 ± 0.08 g after foliar treatment with 1 mM ASA (Table 2). The differences among treatments were assessed statistically using two-way ANOVA followed by DMRT comparisons; the means with different letters significantly differ at the *p* < 0.05 level. The shoot dry weight also increased from 0.64 ± 0.01 g in the non-ASA-treated *P. vulgaris* seedlings to 1.25 ± 0.01 g after foliar treatment with 1 mM ASA (Table 2). The ASA foliar sprays significantly improved the growth of *P. vulgaris* seedlings subjected to chilling stress for 4 days. The shoot FW and DW significantly (*p* < 0.05) increased. Maximum shoot FW and DW values of 2.48 ± 0.002 and 1.72 ± 0.01 g, respectively, were recorded following foliar treatment with 1 mM ASA. The ASA foliar sprays significantly rescued the growth and photosynthetic pigments of *P. vulgaris* seedlings under different chilling stress levels (2 and 4 days).

**Fig. 1:**
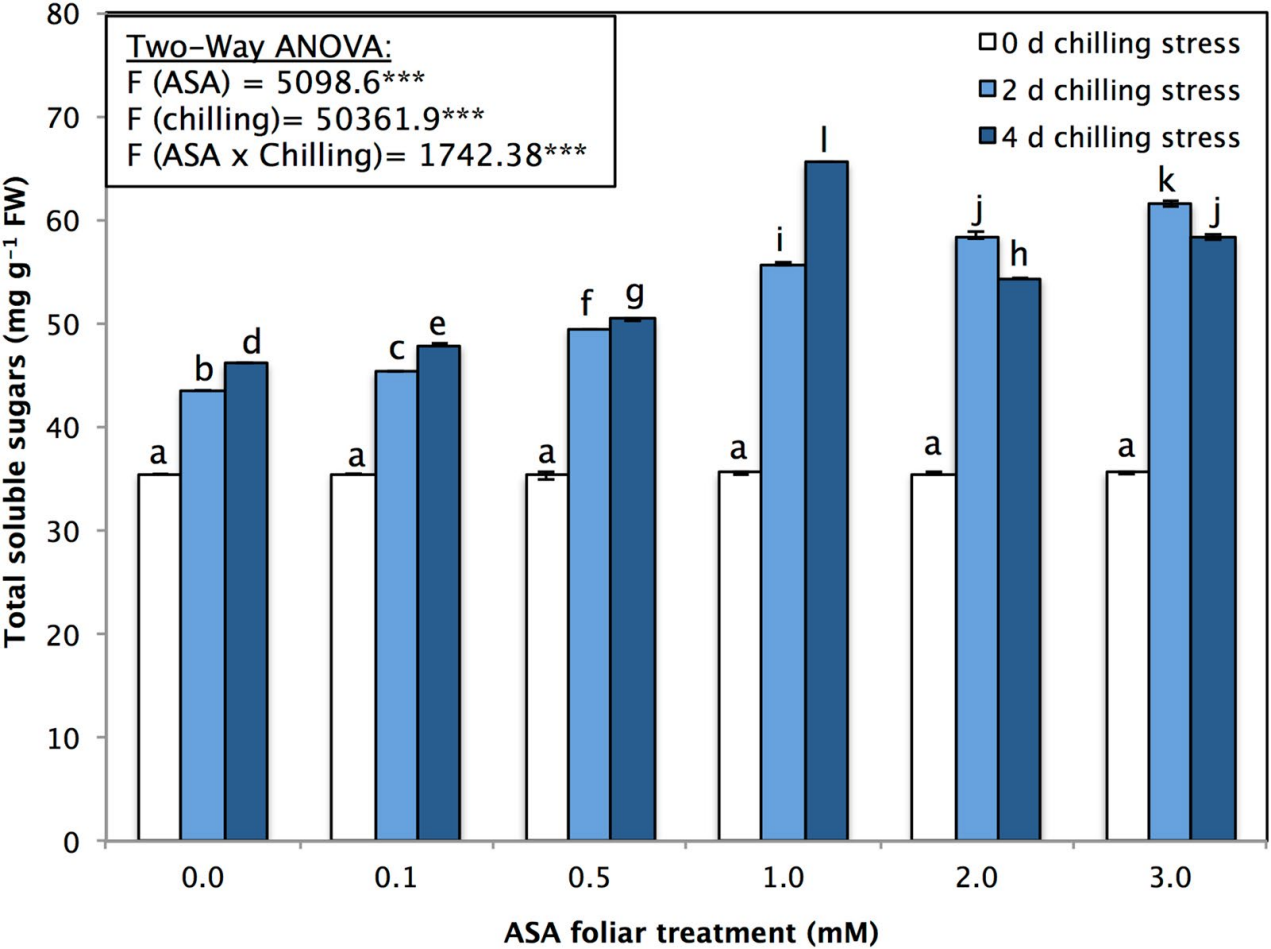
Total soluble sugars (mg g^−1^ FW) of *P. vulgaris* L. after foliar application of different concentration of acetylsalicylic acid (ASA; 0, 0.1, 0.5, 1, 2, 3 mM) and exposed to different level of chilling stress (0, 2, 4 days). Data represented are mean of two replicates ± standard deviation. Variations between different chilling stresses and foliar ASA concentration were assessed by univariate analyses followed by post hoc analysis. Means with the same letters are not significantly different according to Duncan’s multiple comparisons

The carbohydrate metabolism in *P. vulgaris* seedlings was assessed with respect to the total soluble sugar content (mg g^−1^ FW). The total soluble sugars significantly (F_(chilling)_ = 50,361.9; *p* < 0.05) increased between 0 and 4 days of chilling stress in seedlings treated with all concentrations of foliar-sprayed ASA (Fig. 1). The total soluble sugars in the non-stressed *P. vulgaris* seedlings did not significantly (*p* > 0.05) change after being treated with different concentrations of foliar-sprayed ASA (Fig. 1). However, compared with that in non-stressed *P. vulgaris* control seedlings, the total soluble sugar content in *P. vulgaris* seedlings subjected to 2 or 4 days of chilling stress significantly increased in response to all concentrations of ASA foliar sprays. Foliar treatment with ASA significantly increased the total soluble sugars; the 1 mM concentration especially increased the soluble sugars from 35.55 ± 0.18 in non-stressed plants to 55.67 ± 0.073 or 65.47 ± 0.0028 mg g^−1^ FW after 2 or 4 days of chilling stresses (Fig. 1).

**Fig. 2:**
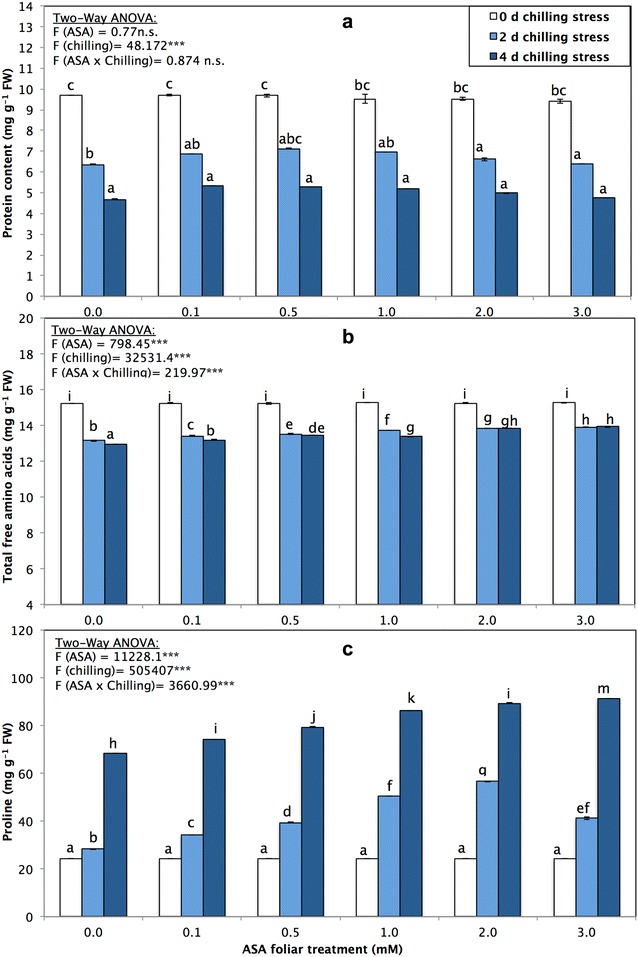
**a** Protein content (mg g^−1^ FW), **b** total free amino acids (mg g^−1^ FW), **c** proline (mg g^−1^ FW) of *P. vulgaris* L. after foliar application of different concentration of acetylsalicylic acid (ASA; 0, 0.1, 0.5, 1, 2, 3 mM) and exposed to different level of chilling stress (0, 2, 4 days). Data represented are mean of two replicates ± standard deviation. Variations between different chilling stresses and foliar ASA concentration were assessed by univariate analyses followed by post hoc analysis. Means with the same letters are not significantly different according to Duncan’s multiple comparisons

Chilling stress induced a significant (*p* < 0.05) decrease in total protein and free amino acid contents in *P. vulgaris* seedlings (Fig. 2a). The total protein content was 9.69 ± 0.007, 6.76 ± 0.006 and 4.67 ± 0.019 mg g^−1^ FW in non-ASA-sprayed seedlings subjected to chilling stress for 0, 2 and 4 days, respectively; the total free amino acids in the non-ASA-sprayed seedlings reached levels of 15.25 ± 0.014, 13.15 ± 0.028 and 12.95 ± 0.014 under the same chilling stress durations; respectively. The treatment of *P. vulgaris* with different concentrations of foliar-sprayed ASA did not induce significant changes in either total protein content or total free amino acids (Fig. 2a, b). However, the protein content of seedlings slightly increased after they were treated with foliar sprays with certain concentrations of ASA. The maximum increase in protein content was recorded in the seedlings treated with 0.5 mM ASA; the protein contents reached 7.13 ± 0.013 and 5.27 ± 0.001 mg g^−1^ FW in the seedlings subjected to chilling stress for 2 and 4 days, respectively. However, the maximum levels of free amino acids were 13.93 ± 0.00 and 13.93 ± 0.02 mg g^−1^ FW in the seedlings subjected to chilling stress for 2 and 4 days, respectively, following treatment with 3 mM ASA (Fig. 2b).

Proline metabolism, in terms of proline content (mg g^−1^ FW), was monitored in the *P. vulgaris* seedlings (Fig. 2c). Proline accumulated significantly (*p* < 0.05) in the cells of *P. vulgaris* seedlings not treated with ASA in response to chilling stress. The proline accumulation increased significantly (*p* < 0.05) from 24.36 ± 0.011 mg g^−1^ FW in the non-stressed seedlings to 28.40 ± 0.20 and 68.51 ± 0.068 mg g^−1^ FW in those subjected to chilling stress for 2 and 4 days (Fig. 2c). However, treatment with ASA significantly (*p* < 0.05) increased the accumulation of proline in *P. vulgaris*, e.g., treatment with 3 mM ASA increased the accumulation from 24.35 ± 0.005 mg g^−1^ FW in the non-stressed seedlings to 41.16 ± 0.44 and 91.35 ± 0.007 mg g^−1^ FW in the seedlings subjected to chilling stress for 2 and 4 days (Fig. 2c).

**Fig. 3:**
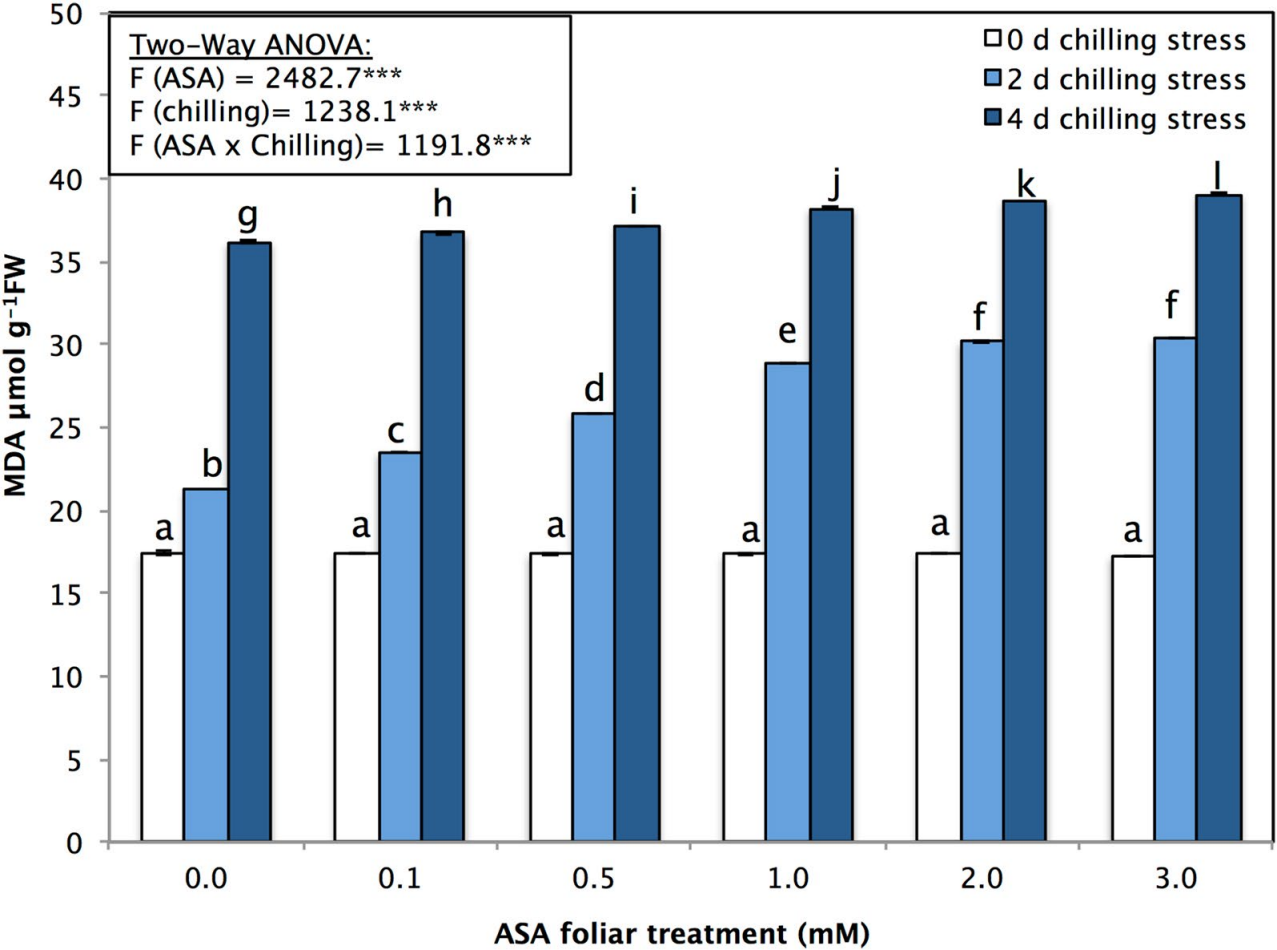
Cellular lipid peroxidation in terms of MDA (μmol g^−1^ FW) of *P. vulgaris* L. after foliar application of different concentration of acetylsalicylic acid (ASA; 0, 0.1, 0.5, 1, 2, 3 mM) and exposed to different level of chilling stress (0, 2, 4 days). Data represented are mean of two replicates ± standard deviation. Variations between different chilling stresses and foliar ASA concentration were assessed by univariate analyses followed by post hoc analysis. Means with the same letters are not significantly different according to Duncan’s multiple comparisons

Cellular lipid peroxidation in terms of MDA was monitored. This method is well recognized to reflect oxidative damage caused by chilling stress. Chilling stress significantly increased cellular lipid peroxidation both in seedlings treated and not treated with ASA (Fig. 3). Compared with the non-stressed control *P. vulgaris* seedlings, the non-stressed ASA-treated *P. vulgaris* seedlings had significantly higher MDA levels under chilling stress and displayed levels of 30.35 ± 0.07 and 39.05 ± 0.070 μmol g^−1^ FW under chilling stress of 2 and 4 days, respectively, following treatment with 3 mM ASA (Fig. 3). The MDA concentrations increased as the duration of low-temperature stress increased during the experiment (Fig. 3). Moreover, the MDA contents in non-stressed seedlings were slightly affected by ASA applications. However, these contents significantly increased in seedlings exposed to different durations of chilling stress (Fig. 3).

**Fig. 4:**
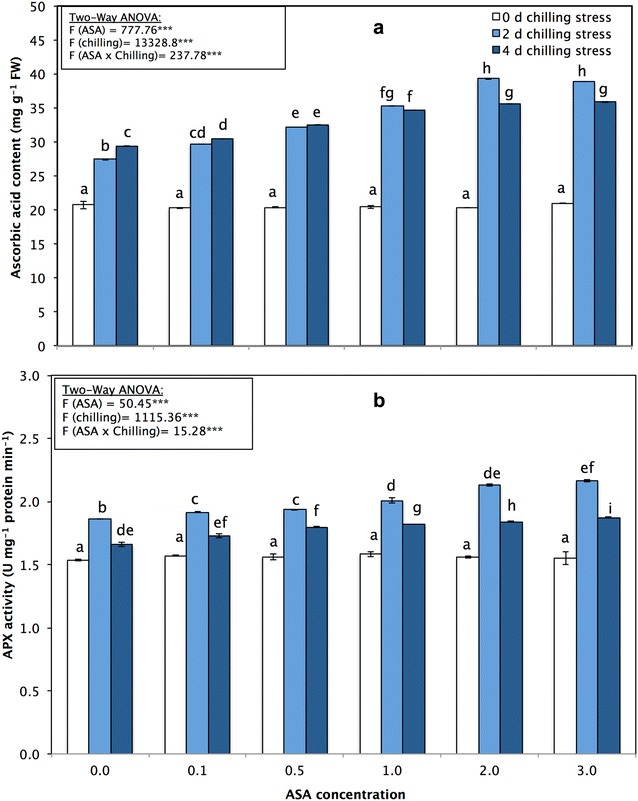
**a** Ascorbic acid content (mg g^−1^ FW) and **b** ascorbic peroxidase (APX; mmol ascorbate min^−1^ g^−1^ FW) in leaves of *P. vulgaris* L. after foliar application of different concentration of acetylsalicylic acid (ASA; 0, 0.1, 0.5, 1, 2, 3 mM) and exposed to different level of chilling stress (0, 2, 4 days). Data represented are mean of two replicates ± standard deviation. Variations between different chilling stresses and foliar ASA concentration were assessed by univariate analyses followed by post hoc analysis. Means with the same letters are not significantly different according to Duncan’s multiple comparisons

The content of AA, a non-enzymatic antioxidant, increased significantly (*p* < 0.0.5) during the experiment and peaked at 39.33 ± 0.14 mg g^−1^ FW after 2 mM ASA and 2 days of chilling stress. The highest level of increase in AA at 4 days of chilling stress was 35.90 ± 0.11 mg g^−1^ FW, recorded after 3 mM ASA treatment (Fig. 4a).

**Fig. 5:**
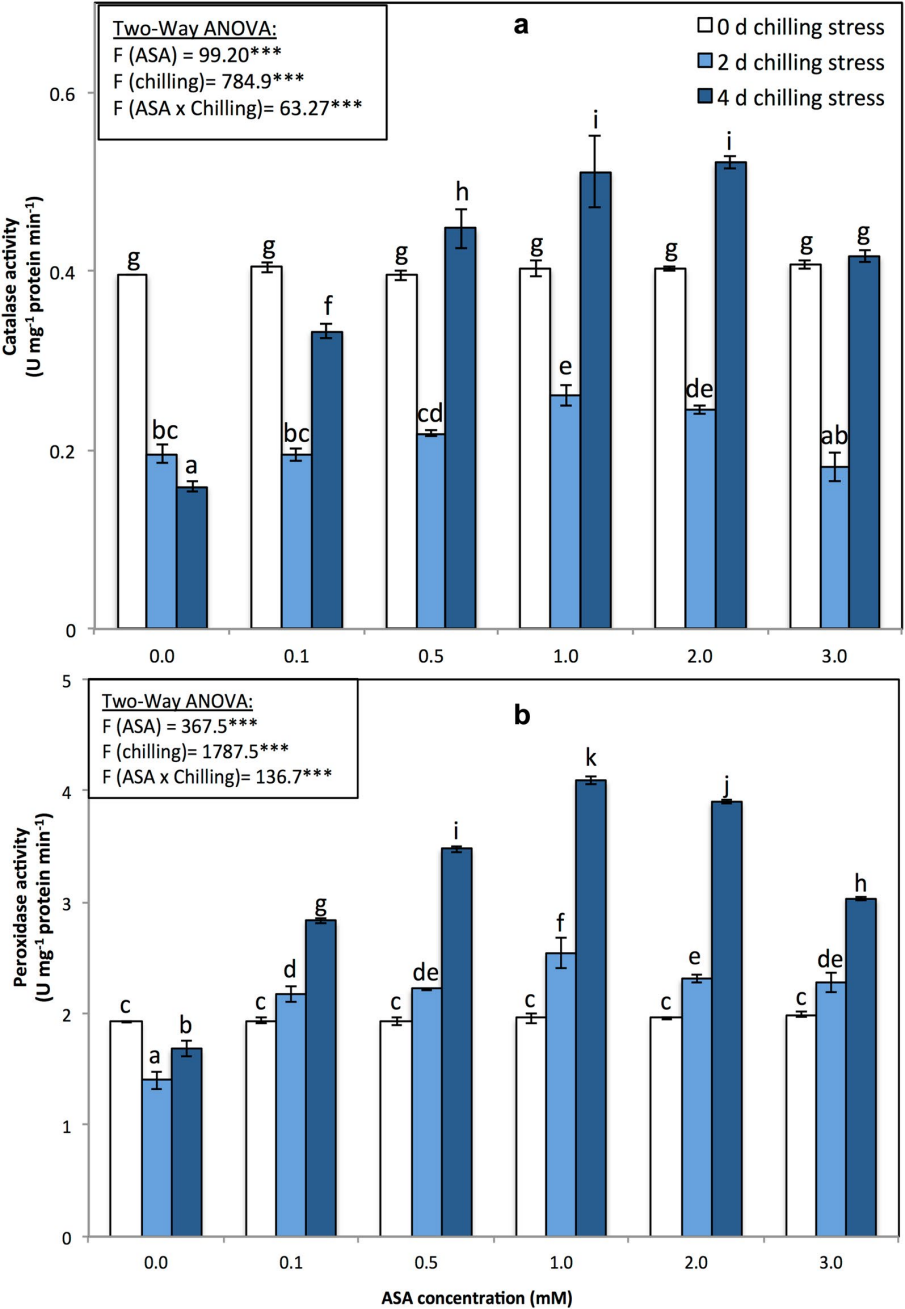
**a** Catalase (unit mg^−1^ protein min^−1^) and **b** peroxidase activities (unit mg^−1^ protein min^−1^) of *P. vulgaris* L. after foliar application of different concentration of acetylsalicylic acid (ASA; 0, 0.1, 0.5, 1, 2, 3 mM) and exposed to different level of chilling stress (0, 2, 4 days). Data represented are mean of two replicates ± standard deviation. Variations between different chilling stresses and foliar ASA concentration were assessed by univariate analyses followed by post hoc analysis. Means with the same letters are not significantly different according to Duncan’s multiple comparisons

**Fig. 6:**
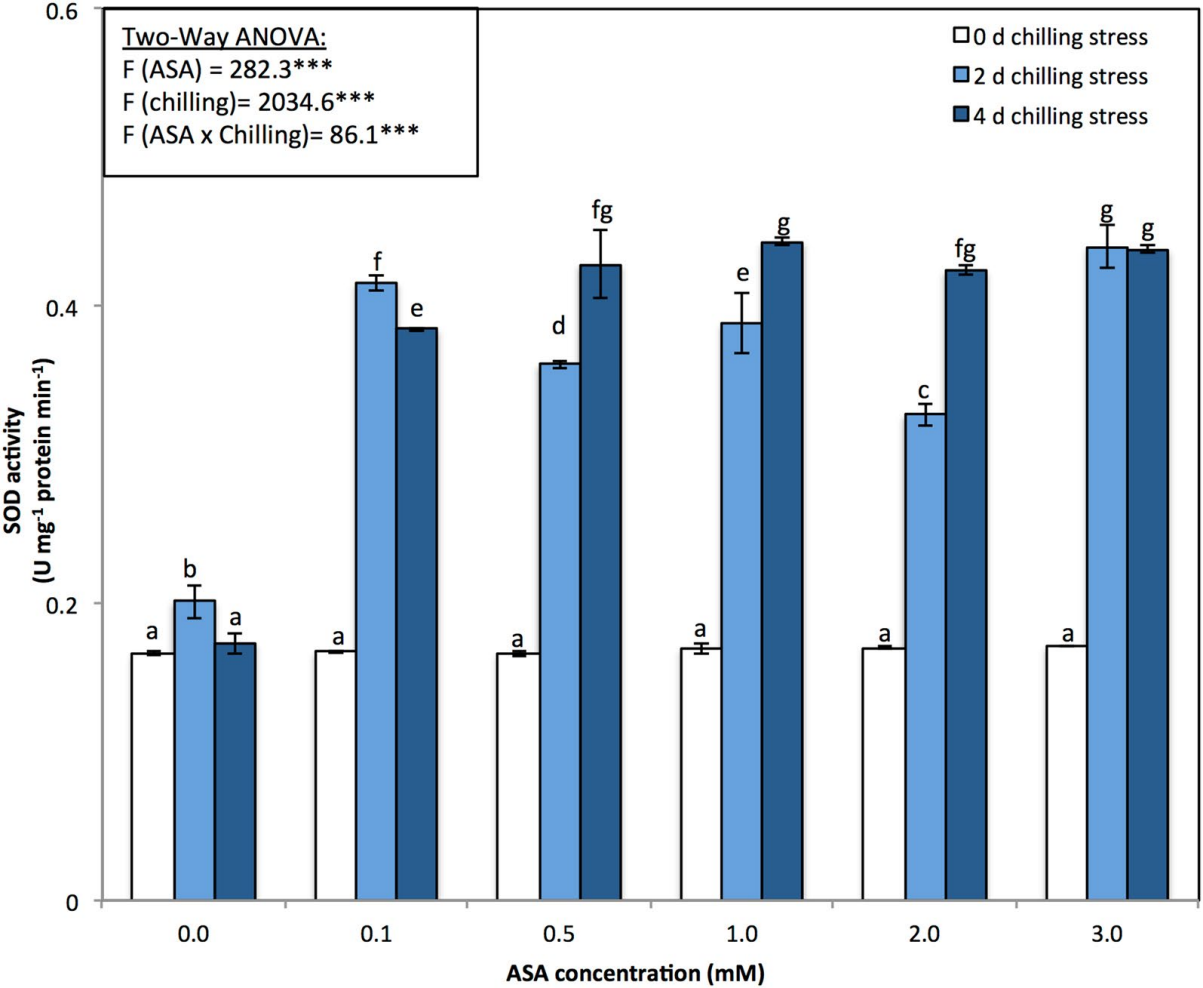
Superoxide dismutase activities (unit mg^−1^ protein min^−1^) of *P. vulgaris* L. after foliar application of different concentration of acetylsalicylic acid (ASA; 0, 0.1, 0.5, 1, 2, 3 mM) and exposed to different level of chilling stress (0, 2, 4 days). Data represented are mean of two replicates ± standard deviation. Variations between different chilling stresses and foliar ASA concentration were assessed by univariate analyses followed by post hoc analysis. Means with the same letters are not significantly different according to Duncan’s multiple comparisons

The enzymatic activities of four antioxidant enzymes, namely, APX, SOD, CAT, and POD, were also monitored in all the experimental variants (Figs. 4b, 5, 6). The activities of the antioxidant enzymes APX, POD, and SOD significantly (*p* < 0.05) increased in response to the different levels of chilling stress; however, CAT activity decreased (Figs. 4b, 5, 6). The activities of the antioxidant enzymes APX, SOD, POD, and CAT significantly increased in response to the application of exogenous ASA, alleviating the adverse effects of chilling stress.

**Fig. 7:**
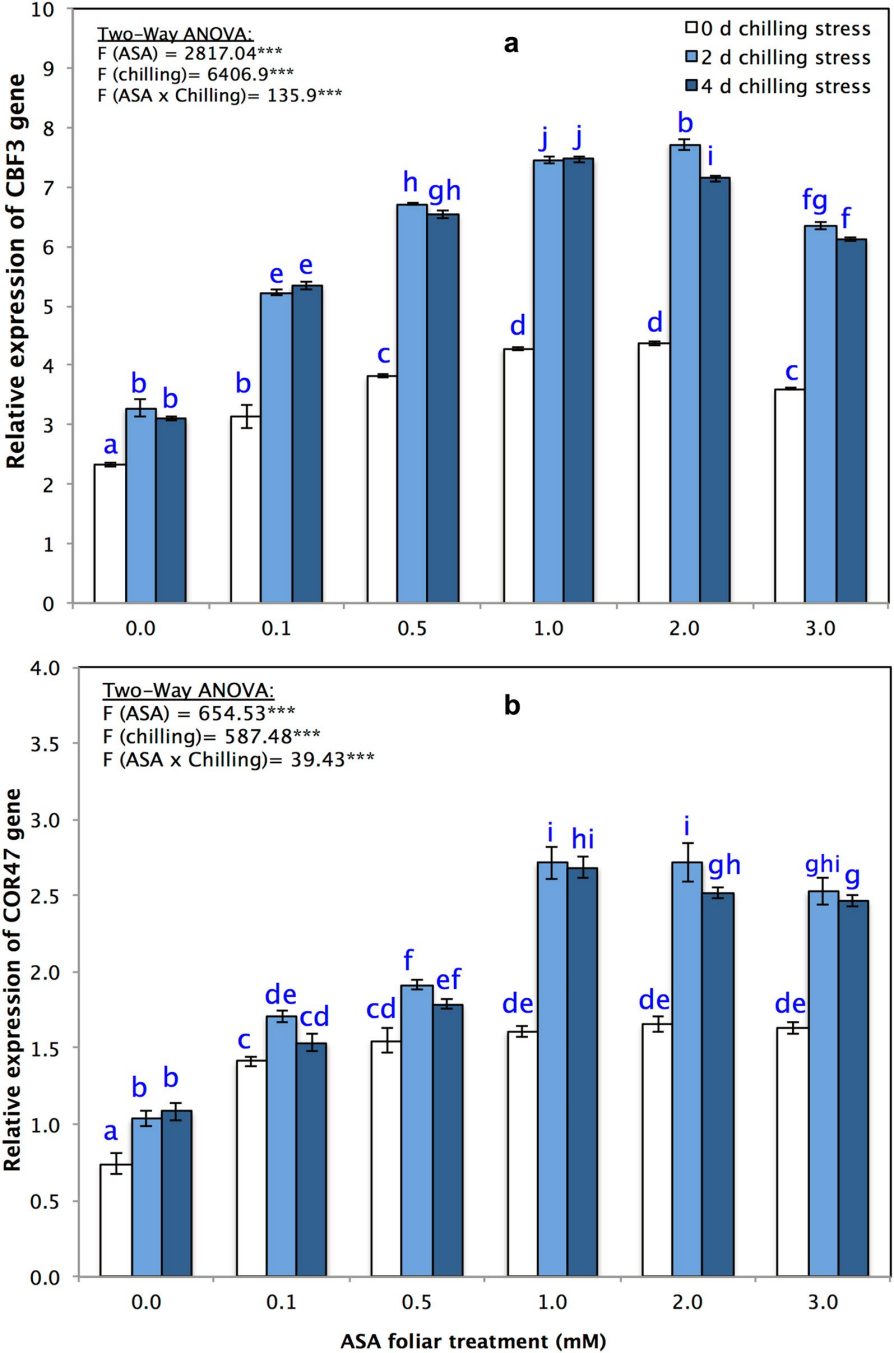
Relative expression of genes **a** CBF3, and **b** COR47 of *P. vulgaris* L. after foliar application of different concentration of acetylsalicylic acid (ASA; 0, 0.1, 0.5, 1, 2, 3 mM) and exposed to a different level of chilling stress (0, 2, 4 days). Data represented are mean of two replicates ± standard deviation. Variations between different chilling stresses and foliar ASA concentration were assessed by univariate analyses followed by post hoc analysis. Means with the same letters are not significantly different according to Duncan’s multiple comparisons

With respect to the qRT-PCR analysis results, the fluorescence intensity of each clone was divided by its corresponding control, after which the relative mRNA levels of CBF3 and COR47 after 0, 2 and 4 days of chilling stress were quantified using RT-qPCR for the seedlings in each of the ASA concentration treatments (0.1, 0.5, 1, 2 and 3 mM). The CBF3 gene expression results presented in Fig. 7a that, compared with the CBF3 gene expression in the control treatment at 0 days (0.81-fold), the maximum expression of the CBF3 gene of 7.72 ± 0.10 was recorded in response to the 2 mM ASA concentration after 2 days (7.72-fold), followed by the 1 mM ASA concentration after 4 days (7.47-fold), the 1 mM ASA concentration after 2 days (7.46-fold), the 0.5 mM ASA concentration after 2 days (6.72-fold), the 0.5 mM ASA concentration after 4 days (6.54-fold) and the 3 mM ASA concentration after 2 days (6.35-fold). The COR47 gene expression results (Fig. 7b) revealed that, compared with the COR47 gene expression levels of the control treatment after 0 days (0.69-fold), the maximum expression levels of the COR47 gene were recorded in response to the 2 mM ASA concentration after 2 days (2.72-fold), followed by the 1 mM ASA concentration after 2 days (2.72-fold), the 1 mM ASA concentration after 4 days (2.69-fold), the 2 mM ASA concentration after 4 days and the 3 mM ASA concentration after 2 days.

The application of exogenous ASA alleviated the adverse effects of chilling stresses for all measured parameters, and the strongest effects were observed in response to 1 and 2 mM ASA. As such, pretreatment with ASA appeared to induce chilling tolerance.

## Discussion

Low-temperature or chilling stress tolerance is a critical feature for economic crops that originate in tropical or temperate regions worldwide. ASA and other phenolic compounds (e.g., SA and benzoic acid) can enhance the chilling tolerance of plants (Kang and Saltveit 2001; Kang et al. 2003; Chinnusamy et al. 2007).

The current study revealed that all growth characteristics and photosynthetic pigments of *P. vulgaris* increased as ASA concentrations increased to a certain level during the chilling stress conditions and that the most significant reduction was observed in the control plants. The most adverse effects of chilling stress observed in the untreated seedlings included reductions in FW, DW, the shoot: root ratio and chlorophyll content (Table 2). The ASA treatment improved the growth characteristics to a certain level under chilling stress; ASA, therefore, acts as a growth stimulant. These effects of ASA application were in agreement with the findings of Senaratna et al. (2000), who reported that 100% of tomato and bean seedlings treated with 0.5 mM ASA survived under chilling stress, while the control plants did not. Moreover, Gharib and Hegazi (2010) reported that soaking *P. vulgaris* seeds in 0.1 mM SA solution also significantly enhance the germination rate, germination percentage and seedling trait under standard and low-temperature stress conditions. Similarly, different morphological and growth criteria of tomato plants treated with foliar applications of salicylaldehyde were enhanced (Kord and Hathout 1992).

Low temperatures cause an imbalance between light harvest and energy use; in turn, the superoxide anion is generated, inducing oxidative stress in chloroplasts (Einset et al. 2007; Gill and Tuteja 2010). ROS can damage the photosynthetic apparatus through the disarrangement of thylakoid structures, suppression of chloroplastic enzymes, and inhibition of D1 protein biosynthesis; the D1 protein is needed for the PSII repair process (Gururani et al. 2015; Choudhury et al. 2017). The exogenous application of ASA most likely significantly increases the activity of the antioxidant system, directly impacting the intensity of photosynthesis. Li and his co-workers (2014) reported that SA significantly enhances *Torreya grandis* biomass under salinity stress due to the improvement in chlorophyll content and the flow of antioxidant enzyme activity that consequently alleviates oxidative stress and enhances the photosynthetic process. Furthermore, the exogenous application of SA effectively prevents iron deficiency, which is the primary cause of plant chlorosis (Kong et al. 2014).

Moreover, changes in total soluble sugars (Fig. 1) and carbohydrate metabolism act as energy factors required for driving acclimation under chilling stress (Gusta and Wisniewski 2013). By interacting with the lipid bilayer, sucrose and other sugars (as compatible solutes) significantly affect plant freezing tolerance and the protection of cellular membranes from damage (Hansen et al. 1997; Liu et al. 1998; Shalaev and Steponkus 2001; Frankow-Lindberg 2001; Arroyo et al. 2003; Shao et al. 2007). The accumulation of soluble sugars contributes to increased membrane cryostability, which is a prerequisite for freezing tolerance in plants (Arroyo et al. 2003; Shao et al. 2007; Gusta and Wisniewski 2013). In agreement with the findings of Nafeh and Hegazi (2009), the present study showed that ASA treatment improved plant tolerance against chilling stress and, compared with those under normal conditions, significantly increases in soluble sugars content. Under stress conditions, increases in soluble sugars lead to enhanced resistance against water loss, the protection of chloroplasts and the acceleration of plant growth (Khodary 2004; Kader et al. 2011; Fayez and Bazaid 2014).

ASA has a protective role. It neutralizes free radicals induced by chilling stress and prevents the destruction of proteins, and in the present study, it increased the amount of protein in treated seedlings (Shinozaki et al. 2003). In response to chilling stress, plants lower the osmotic potential of the cytosol by synthesizing and accumulating compatible solutes and by synthesizing cold acclimation-induced proteins, all of which trigger crop tolerance to chilling stress (Frankow-Lindberg 2001; Murakeözy et al. 2003; Yancey 2005).

Free proline is involved in plant resistance and accumulates under several stress conditions (Parvanova et al. 2004; Demiral and Turkan 2005; Awasthi et al. 2015). In our findings, the proline content significantly increased in the chilling-stressed seedlings of *P. vulgaris* treated with different levels of ASA (Fig. 2). Our data suggest that proline plays a role in the protection against chilling stress by assisting osmotic regulation, removing hydroxyl free radicals and avoiding the destruction of enzymes (Kuznetsov and Shevyakova 1999). The accumulation of free proline also constitutes a vital element of tolerance in other species (Barka et al. 2006; Apostolova et al. 2008; Esra et al. 2010). Treatment with 0.5 mM SA mitigates heat stress by increasing proline production by increasing γ-glutamyl kinase and decreasing proline oxidase activity, resulting in the promotion of osmotic potential necessary for maintaining crucial physiological processes (Khan et al. 2013).

Measurements of MDA are usually used to assess the extent of membrane damage caused by chilling stress. The present study found that, compared with the control applications, exogenous applications of ASA failed to reduce the severity of membrane damage (Fig. 3). Mittler et al. (2004) proposed that membrane damage might be caused by high H_2_O_2_ levels, which could accelerate the Haber–Weiss reaction, resulting in hydroxyl radical formation and thus lipid peroxidation.

The total AA concentrations increased markedly in ASA-treated common bean seedlings as well as in those subjected to chilling stress (Fig. 4). This increase may occur because the AA proved to be a ubiquitous compound that effectively boosts stress tolerance in plants (Miguel et al. 2006; Khan et al. 2012; Naz et al. 2016). AA have a major role during the minimization of ROS activity by enzymatic and non-enzymatic detoxification (Mittler et al. 2004; Borland et al. 2006; Shao et al. 2007). Our findings are in line with those of Kader et al. (2011), who reported that the ascorbate content increased in wheat plants under chilling stress. Our results agree with those of Tirani et al. (2013), who confirmed that SA induces an increase in the AA content in canola plants; those authors suggested that the increase in AA occurs because SA affects the pathways of AA biosynthesis.

Chilling stress triggers the formation of ROS, which in turn leads to stronger oxidative stress in plants. However, ROS can be classified as signaling molecules that regulate plant development and responses to biotic and abiotic stresses (Apel and Hirt 2004; Mittler et al. 2004; Perez and Brown 2014). In our research, the SOD, POD and APX enzyme activities significantly increased during chilling stress and in response to foliar applications of ASA, while the CAT enzyme activity decreased (Figs. 4, 5). The enhancement of antioxidant enzyme activity under chilling stress has been reported in many plant species (Hodges et al. 1996; Kratsch and Wise 2000; Bracale and Coraggio 2003; Einset et al. 2007; Zhao et al. 2013; Ruelland 2017). When applied at low concentrations, SA causes transient oxidative stress in plants, which acts as a hardening process, increasing the antioxidant capacity of plants (Wang et al. 2003; Horváth et al. 2007). Exogenous applications of SA can prevent isozyme activity, including that of CAT-1 and CAT-2, which in turn can mediate responses to low-temperature stress in *Z. may*s plants (Horváth et al. 2007). In the present study, by inducing the activity of APX, POX, and SOD, ASA strongly reduced the effects of chilling stress on all parameters measured in the plants. Similar results were also reported by Mutlu et al. (2013) and He and Zhu (2008) in wheat and tomato, respectively. Positive correlations among SOD, POD and APX activities suggest that increased SOD activity is accompanied by increases in POD and APX activities because of the high demand from H_2_O_2_ quenching (Senaratna et al. 2000).

Low-temperature stress triggers the expression of chilling stress-associated genes. Among several cold signaling pathways, the involvement of *CBF3* and *COR47* is critical in plant chilling tolerance and cold acclimation (Miura and Furumoto 2013). Our results demonstrate that the CBF3 and COR47 genes exhibit a significant increase in their relative expression level (fold change) in accordance with the application of different ASA concentrations to plants under chilling stress (Fig. 7a, b). Chilling stress amplified the expression of the tested CBF3 genes in the *P. vulgaris* seedlings (Fig. 7a), suggesting a functional similarity of CBFs in *P. vulgaris* as well as Arabidopsis in response to low-temperature stress (Gilmour et al. 2004; Thomashow 2010). Application of ASA under chilling stress enhanced the expression of CBF3 together with the COR47 gene (Fig. 7b). In Arabidopsis, three CBF/DREB1 proteins take part in the control of COR gene expression and chilling tolerance (Gilmour et al. 2000, 2004). A calmodulin-binding transcription activator, CAMTA3/AtSR1, identifies the promoter of CBF2/DREB1C to upregulate genes associated with chilling tolerance, indicating that SA signaling and low-temperature signaling are interconnected (Du et al. 2009; Doherty et al. 2009).

Overexpression of *CfCBF3* increases chilling tolerance and causes no dwarf phenotype (Hanin et al. 2011). This overexpression also leads to multiple biochemical and physiological changes associated with chilling stress. Higher contents of proline and soluble sugars and lower contents of ROS have been observed in transgenic plants (Yang et al. 2011; Miura and Furumoto 2013).

## Conclusions

Chilling temperatures are responsible for a range of physiological disturbances in chilling-sensitive plants and can cause chilling injury and death of many horticulture plants such as *P. vulgaris*. In this study, the chilling tolerance of *P. vulgaris* could be significantly improved by the exogenous application of ASA. The physiological and molecular data ultimately revealed that ASA could mutually induce and maintain homeostasis to exert synergistic effects on common bean plant chilling stress. The present study confirmed that the optimum concentrations of ASA for alleviating the effects of chilling stress proved to be 1 and 2 mM ASA, as maximum stimulation of the antioxidant enzyme system occurred in response to these concentrations. These results indicate that ASA can effectively be used to protect *P. vulgaris* from the damaging effects of chilling stress during the early stages of growth.

## Abbreviations

AA: ascorbic acid
ASA: acetylsalicylic acid
APX: ascorbate peroxidase
BSA: bovine serum albumin
CAT: catalase
DAC: days after chilling
DMRTs: Duncan multiple range tests
FW: fresh weight
MDA: malondialdehyde
PPFD: photosynthetic photon flux density
POD: peroxidase
ROS: reactive oxygen species
SOD: superoxide dismutase

## References

[CR1] AbdElgawad H, Zinta G, Hegab MM (2016). High salinity induces different oxidative stress and antioxidant responses in maize seedlings organs. Front Plant Sci.

[CR2] Aebi H (1984) [13] Catalase in vitro. In: Packer L (ed) Methods in enzymology. Academic Press, New York, pp 121–126 10.1016/s0076-6879(84)05016-3 6727660

[CR3] Allen DJ, Ort DR (2001). Impacts of chilling temperatures on photosynthesis in warm-climate plants. Trends Plant Sci.

[CR4] Apel K, Hirt H (2004). Reactive oxygen species: metabolism, oxidative stress, and signal transduction. Annu Rev Plant Biol.

[CR5] Apostolova P, Yordanova R, Popova L (2008). Response of antioxidative defence system to low temperature stress in two wheat cultivars. Gen Appl Plant Physiol.

[CR6] Arroyo A, Bossi F, Finkelstein RR, León P (2003). Three genes that affect sugar sensing (abscisic acid insensitive 4, abscisic acid insensitive 5, and constitutive triple response 1) are differentially regulated by glucose in Arabidopsis. Plant Physiol.

[CR7] Awasthi R, Bhandari K, Nayyar H (2015). Temperature stress and redox homeostasis in agricultural crops. Front Environ Sci.

[CR8] Barka EA, Nowak J, Clement C (2006). Enhancement of chilling resistance of inoculated grapevine plantlets with a plant growth-promoting rhizobacterium, *Burkholderia phytofirmans* strain PsJN. Appl Environ Microbiol.

[CR9] Bita CE, Gerats T (2013). Plant tolerance to high temperature in a changing environment: scientific fundamentals and production of heat stress-tolerant crops. Front Plant Sci.

[CR10] Borland A, Elliott S, Patterson S (2006). Are the metabolic components of crassulacean acid metabolism up-regulated in response to an increase in oxidative burden?. J Exp Bot.

[CR11] Bracale M, Coraggio I (2003) Chilling and freezing stresses in plants: cellular responses and molecular strategies for adaptation. In: di Toppi LS, Pawlik-Skowrońska B (eds) Abiotic stresses in plants. Springer, Dordrecht, pp 23–51

[CR12] Bradford MM (1976). A rapid and sensitive method for the quantitation of microgram quantities of protein utilizing the principle of protein-dye binding. Anal Biochem.

[CR13] Caffagni A, Pecchioni N, Francia E (2014). Candidate gene expression profiling in two contrasting tomato cultivars under chilling stress. Biol Plant.

[CR14] Canakci S, Munzuroğlu O (2007). Effects of acetylsalicylic acid on germination, growth and chlorophyll amounts of cucumber (*Cucumis sativus* L.) seeds. Pak J Biol Sci PJBS.

[CR15] Chartzoulakis K, Psarras G (2005). Global change effects on crop photosynthesis and production in Mediterranean: the case of Crete, Greece. Agric Ecosyst Environ.

[CR16] Chinnusamy V, Zhu J, Zhu JK (2007). Cold stress regulation of gene expression in plants. Trends Plant Sci.

[CR17] Choudhury FK, Rivero RM, Blumwald E, Mittler R (2017). Reactive oxygen species, abiotic stress and stress combination. Plant J.

[CR18] Ciha AJ, Brun WA (1978). Effect of pod removal on nonstructural carbohydrate concentration in soybean tissue. Crop Sci.

[CR19] Cramer GR, Urano K, Delrot S (2011). Effects of abiotic stress on plants: a systems biology perspective. BMC Plant Biol.

[CR20] De Vos CHR, Schat H, De Waal MAM (1991). Increased resistance to copper-induced damage of the root cell plasmalemma in copper tolerant *Silene cucubalus*. Physiol Plant.

[CR21] Demiral T, Turkan I (2005). Comparative lipid peroxidation, antioxidant defense systems and proline content in roots of two rice cultivars differing in salt tolerance. Environ Exp Bot.

[CR22] Doherty CJ, Van Buskirk HA, Myers SJ, Thomashow MF (2009). Roles for Arabidopsis CAMTA transcription factors in cold-regulated gene expression and freezing tolerance. Plant Cell.

[CR23] Du L, Ali GS, Simons KA (2009). Ca(2+)/calmodulin regulates salicylic-acid-mediated plant immunity. Nature.

[CR24] Dubey RS, Rani M (1989). Influence of NaCl salinity on growth and metabolic status of protein and amino acids in rice seedlings. J Agron Crop Sci.

[CR25] Einset J, Winge P, Bones A (2007). ROS signaling pathways in chilling stress. Plant Signal Behav.

[CR26] El Kelish A, Zhao F, Heller W (2014). Ragweed (*Ambrosia artemisiifolia*) pollen allergenicity: SuperSAGE transcriptomic analysis upon elevated CO_2_ and drought stress. BMC Plant Biol.

[CR27] Esra K, İŞLEK C, Üstün AS (2010). Effect of cold on protein, proline, phenolic compounds and chlorophyll content of two pepper (*Capsicum annuum* L.) varieties. Gazi Univ J Sci.

[CR28] Fayez KA, Bazaid SA (2014). Improving drought and salinity tolerance in barley by application of salicylic acid and potassium nitrate. J Saudi Soc Agric Sci.

[CR29] Foyer CH, Noctor G (2003). Redox sensing and signalling associated with reactive oxygen in chloroplasts, peroxisomes and mitochondria. Physiol Plant.

[CR30] Frankow-Lindberg BE (2001). Adaptation to winter stress in nine white clover populations: changes in non-structural carbohydrates during exposure to simulated winter conditions and “spring” regrowth potential. Ann Bot.

[CR31] Gautam S, Singh PK (2009). Salicylic acid-induced salinity tolerance in corn grown under NaCl stress. Acta Physiol Plant.

[CR32] Gharib FA, Hegazi AZ (2010). Salicylic acid ameliorates germination, seedling growth, phytohormone and enzymes activity in bean (*Phaseolus vulgaris* L.) under cold stress. J Am Sci.

[CR33] Giannopolitis CN, Ries SK (1977). Superoxide dismutases: I. Occurrence in higher plants. Plant Physiol.

[CR34] Gill SS, Tuteja N (2010). Reactive oxygen species and antioxidant machinery in abiotic stress tolerance in crop plants. Plant Physiol Biochem.

[CR35] Gilmour SJ, Sebolt AM, Salazar MP (2000). Overexpression of the Arabidopsis CBF3 transcriptional activator mimics multiple biochemical changes associated with cold acclimation. Plant Physiol.

[CR36] Gilmour SJ, Fowler SG, Thomashow MF (2004). Arabidopsis transcriptional activators CBF1, CBF2, and CBF3 have matching functional activities. Plant Mol Biol.

[CR37] Gupta N, Rathore M, Goyary D (2012). Marker-free transgenic cucumber expressing Arabidopsis cbf1 gene confers chilling stress tolerance. Biol Plant.

[CR38] Gururani MA, Venkatesh J, Tran LSP (2015). Regulation of photosynthesis during abiotic stress-induced photoinhibition. Mol Plant.

[CR39] Gusta LV, Wisniewski M (2013). Understanding plant cold hardiness: an opinion. Physiol Plant.

[CR40] Guy CL (1990). Cold acclimation and freezing stress tolerance: role of protein metabolism. Annu Rev Plant Physiol Plant Mol Biol.

[CR41] Hanin M, Brini F, Ebel C (2011). Plant dehydrins and stress tolerance. Plant Signal Behav.

[CR42] Hansen J, Turkington R, Vogg G et al (1997) Conifer carbohydrate physiology: updating classical views. In: Rennenberg H, Eschrich W, Ziegler H (eds.) Trees—contributions to modern tree physiology. Backhuys, Leiden, pp 97–108

[CR43] He Y, Zhu ZJ (2008). Exogenous salicylic acid alleviates NaCl toxicity and increases antioxidative enzyme activity in *Lycopersicon esculentum*. Biol Plant.

[CR44] Hodges DM, Andrews CJ, Johnson DA, Hamilton RI (1996). Antioxidant compound responses to chilling stress in differentially sensitive inbred maize lines. Physiol Plant.

[CR45] Holder M, Goodwin TW (1965). Chlorophylls: chemistry and biochemistry of plant pigments. Chemistry and biochemistry of plant pigments.

[CR46] Horváth E, Szalai G, Janda T (2007). Induction of abiotic stress tolerance by salicylic acid signaling. J Plant Growth Regul.

[CR47] Irigoyen JJ, Einerich DW, Sanchez-Diaz M (1992). Water stress induced changes in concentrations of proline and total soluble sugars in nodulated alfalfa (*Medicago sativa*) plants. Physiol Plant.

[CR48] Jaglo KR, Kleff S, Amundsen KL (2001). Components of the Arabidopsis C-repeat/dehydration-responsive element binding factor cold-response pathway are conserved in *Brassica napus* and other plant species. Plant Physiol.

[CR49] Jaleel CA, Manivannan P, Wahid A (2009). Drought stress in plants: a review on morphological characteristics and pigments composition. Int J Agric Biol.

[CR50] Jiang AL, Tian S, Xu Y (2002). Effect of controlled atmospheres with high O_2_ or high-CO_2_ concentrations on postharvest physiology and storability of “Napoleon” sweet cherry. Acta Bot Sin.

[CR51] Kader DZA, Saleh AAH, Elmeleigy SA, Dosoky NS (2011). Chilling-induced oxidative stress and polyamines regulatory role in two wheat varieties. J Taibah Univ Sci.

[CR52] Kang H-M, Saltveit ME (2001). Activity of enzymatic antioxidant defense systems in chilled and heat shocked cucumber seedling radicles. Physiol Plant.

[CR53] Kang G, Wang C, Sun G, Wang Z (2003). Salicylic acid changes activities of H_2_O_2_-metabolizing enzymes and increases the chilling tolerance of banana seedlings. Environ Exp Bot.

[CR54] Kasuga M, Liu Q, Miura S (1999). Improving plant drought, salt, and freezing tolerance by gene transfer of a single stress-inducible transcription factor. Nat Biotechnol.

[CR55] Khan MIR, Khan NA (2013). Salicylic acid and jasmonates: approaches in abiotic stress tolerance. J Plant Biochem Physiol.

[CR56] Khan W, Prithiviraj B, Smith DL (2003). Photosynthetic responses of corn and soybean to foliar application of salicylates. J Plant Physiol.

[CR57] Khan NA, Nazar R, Iqbal N, Anjum NA (2012). Phytohormones and abiotic stress tolerance in plants.

[CR58] Khan MIR, Iqbal N, Masood A (2013). Salicylic acid alleviates adverse effects of heat stress on photosynthesis through changes in proline production and ethylene formation. Plant Signal Behav.

[CR60] Khodary SEA (2004). Effect of salicylic acid on the growth, photosynthesis and carbohydrate metabolism in salt-stressed maize plants. Int J Agric Biol.

[CR61] Kong J, Dong Y, Xu L (2014). Effects of foliar application of salicylic acid and nitric oxide in alleviating iron deficiency induced chlorosis of *Arachis hypogaea* L. Bot Stud.

[CR62] Kord M, Hathout T (1992) Changes in some growth criteria, metabolic activities and endogenous hormones in tomato plants consequent to spraying with different concentrations of salicylaldehyde. Egypt J Physiol Sci. https://eurekamag.com/research/002/318/002318832.php. Accessed 20 Sep 2017

[CR63] Kratsch HA, Wise RR (2000). The ultrastructure of chilling stress. Plant Cell Environ.

[CR64] Kupferwasser LI, Yeaman MR, Shapiro SM (1999). Acetylsalicylic acid reduces vegetation bacterial density, hematogenous bacterial dissemination, and frequency of embolic events in experimental Staphylococcus aureus endocarditis through antiplatelet and antibacterial effects. Circulation.

[CR65] Kuznetsov VV, Shevyakova NI (1999). Proline under stress: biological role, metabolism, and regulation. Russ J Plant Physiol.

[CR66] Li YC, Jiang XX, Long XJ (2014). Effects and action mechanisms of sodium fluoride (NaF) on the growth and cephalotaxine production of *Cephalotaxus mannii* suspension cells. Enzyme Microb Technol.

[CR67] Liu Q, Kasuga M, Sakuma Y (1998). Two transcription factors, DREB1 and DREB2, with an EREBP/AP2 DNA binding domain separate two cellular signal transduction pathways in drought- and low-temperature-responsive gene expression, respectively, in Arabidopsis. Plant Cell.

[CR68] Livak KJ, Schmittgen TD (2001). Analysis of relative gene expression data using real-time quantitative PCR and the 2(-Delta Delta C(T)) method. Methods San Diego Calif.

[CR69] Mahajan S, Tuteja N (2005). Cold, salinity and drought stresses: an overview. Arch Biochem Biophys.

[CR70] McHenry EW, Graham M (1935). Observations on the estimation of ascorbic acid by titration. Biochem J.

[CR71] Miguel G, Fontes C, Martins D (2006). Effects of post-harvest treatment and storage time on the organic acid content in Assaria and Mollar pomegranate (*Punica granatum* L.) fruit. Ital J Food Sci.

[CR72] Miller G, Suzuki N, Ciftci-Yilmaz S, Mittler R (2010). Reactive oxygen species homeostasis and signalling during drought and salinity stresses. Plant Cell Environ.

[CR73] Mittler R, Vanderauwera S, Gollery M, Van Breusegem F (2004). Reactive oxygen gene network of plants. Trends Plant Sci.

[CR74] Miura K, Furumoto T (2013). Cold signaling and cold response in plants. Int J Mol Sci.

[CR75] Miura K, Tada Y (2014). Regulation of water, salinity, and cold stress responses by salicylic acid. Front Plant Sci.

[CR76] Movahedi S, Tabatabaei BES, Alizade H (2012). Constitutive expression of Arabidopsis DREB1B in transgenic potato enhances drought and freezing tolerance. Biol Plant.

[CR77] Murakeözy ÉP, Nagy Z, Duhazé C (2003). Seasonal changes in the levels of compatible osmolytes in three halophytic species of inland saline vegetation in Hungary. J Plant Physiol.

[CR78] Mutlu S, Karadağoğlu Ö, Atici Ö, Nalbantoğlu B (2013). Protective role of salicylic acid applied before cold stress on antioxidative system and protein patterns in barley apoplast. Biol Plant.

[CR79] Nafeh A, Hegazi A (2009). Effect of acetylsalicylic acid, indole-3-bytric acid and gibberellic acid on plant growth and yield of pea (*Pisum sativum* L.). Aust J Basic Appl Sci.

[CR80] Naz H, Aisha N, Ashraf M (2016). Impact of ascorbic acid on growth and some physiological attributes of cucumber (Cucumis sativus) plants under water-deficit conditions. Pak J Bot.

[CR81] Parvanova D, Ivanov S, Konstantinova T (2004). Transgenic tobacco plants accumulating osmolytes show reduced oxidative damage under freezing stress. Plant Physiol Biochem PPB.

[CR82] Pereira A (2016). Plant abiotic stress challenges from the changing environment. Front Plant Sci 7.

[CR83] Perez IB, Brown PJ (2014). The role of ROS signaling in cross-tolerance: from model to crop. Front Plant Sci.

[CR84] Rao RP, Yuan C, Allegood JC (2007). Ceramide transfer protein function is essential for normal oxidative stress response and lifespan. Proc Natl Acad Sci.

[CR85] Raskin I (1992). Role of salicylic acid in plants. Annu Rev Plant Physiol Plant Mol Biol.

[CR86] Ruelland E, Shabala S (2017). Plant responses to chilling temperatures. Plant stress physiology.

[CR87] Sadasivam S (1992) Biochemical methods for agricultural sciences. New Age International Pub. (P) Limited

[CR88] Saibo NJM, Lourenço T, Oliveira MM (2009). Transcription factors and regulation of photosynthetic and related metabolism under environmental stresses. Ann Bot.

[CR89] Saleh AAH, Abdel-Kader D, El Kelish A (2007). Role of heat shock and salicylic acid in antioxidant homeostasis in mungbean (*Vigna radiata* L.) plant subjected to heat stress. Am J Plant Physiol.

[CR90] Senaratna T, Touchell D, Bunn E, Dixon K (2000). Acetyl salicylic acid (Aspirin) and salicylic acid induce multiple stress tolerance in bean and tomato plants. Plant Growth Regul.

[CR91] Shalaev EY, Steponkus PL (2001). Phase behavior and glass transition of 1,2-dioleoylphosphatidylethanolamine (DOPE) dehydrated in the presence of sucrose. Biochim Biophys Acta.

[CR92] Shao HB, Chu LY, Lu ZH, Kang CM (2007). Primary antioxidant free radical scavenging and redox signaling pathways in higher plant cells. Int J Biol Sci.

[CR93] Shinozaki K, Yamaguchi-Shinozaki K, Seki M (2003). Regulatory network of gene expression in the drought and cold stress responses. Curr Opin Plant Biol.

[CR94] Simaei M, Khavari-Nejad RA, Bernard F (2012). Exogenous application of salicylic acid and nitric oxide on the ionic contents and enzymatic activities in NaCl-stressed soybean plants. Am J Plant Sci.

[CR95] Strain H, Svec, Vernon LP, Seely GR (1966). Extraction, separation, estimation and isolation of the chlorophylls. The chlorophylls.

[CR96] Tabassum T, Farooq M, Ahmad R (2017). Seed priming and transgenerational drought memory improves tolerance against salt stress in bread wheat. Plant Physiol Biochem.

[CR97] Thomashow MF (1998). Role of cold-responsive genes in plant freezing tolerance. Plant Physiol.

[CR98] Thomashow MF (2001). So what’s new in the field of plant cold acclimation? Lots!. Plant Physiol.

[CR99] Thomashow MF (2010). Molecular basis of plant cold acclimation: insights gained from studying the CBF cold response pathway. Plant Physiol.

[CR100] Tirani MM, Nasibi F, Kalantari KM (2013). Interaction of salicylic acid and ethylene and their effects on some physiological and biochemical parameters in canola plants (*Brassica napus* L.). Photosynthetica.

[CR101] Tong L, DanWei M, WuYuan D, Fang C (2005). Effects of low temperature on physiological indices of Jatropha curcas. Chin J Oil Crop Sci.

[CR102] Wang W, Vinocur B, Altman A (2003). Plant responses to drought, salinity and extreme temperatures: towards genetic engineering for stress tolerance. Planta.

[CR103] Yancey PH (2005). Organic osmolytes as compatible, metabolic and counteracting cytoprotectants in high osmolarity and other stresses. J Exp Biol.

[CR104] Yang S, Tang XF, Ma NN (2011). Heterology expression of the sweet pepper CBF3 gene confers elevated tolerance to chilling stress in transgenic tobacco. J Plant Physiol.

[CR105] Zandalinas SI, Mittler R, Balfagón D (2017). Plant adaptations to the combination of drought and high temperatures. Physiol Plant.

[CR106] Zhao XQ, Wang WS, Zhang F (2013). Temporal profiling of primary metabolites under chilling stress and its association with seedling chilling tolerance of rice (*Oryza sativa* L.). Rice.

